# SAMViTrack: A Search-Region Adaptive Mamba-ViT Tracker for Real-Time UAV Tracking

**DOI:** 10.3390/s25247454

**Published:** 2025-12-07

**Authors:** Xiaoyu Guo, Yian Li, Hao Zhang, Xucheng Wang, Dan Zeng, Feixiang He, Shuiwang Li

**Affiliations:** 1 College of Computer Science and Engineering, Guilin University of Technology, Guilin 541006, China; guoxiaoyu@glut.edu.cn (X.G.); liyian@glut.edu.cn (Y.L.); zhanghao@glut.edu.cn (H.Z.); 2 Guangxi Key Laboratory of Embedded Technology and Intelligent System, Guilin University of Technology, Guilin 541004, China; 3 School of Computer Science, Fudan University, Shanghai 200082, China; xcwang@glut.edu.cn; 4 School of Artificial Intelligence, Sun Yat-sen University, Zhuhai 519000, China; zengd8@mail.sysu.edu.cn; 5 School of Electronic Information, Central South University, Changsha 410083, China; feixiang.he@csu.edu.cn

**Keywords:** Mamba attention, Vision Transformer, object tracking, UAV applications, adaptive search region

## Abstract

Achieving fast and robust object tracking is critical for real-time Unmanned Aerial Vehicle (UAV) applications, where targets often move unpredictably and environmental conditions can rapidly change. In this paper, we propose the Search-Region Adaptive Mamba-ViT Tracker (SAMViTrack), a novel framework that combines the efficiency of Mamba attention with the powerful feature extraction capabilities of Vision Transformer (ViT). Our tracker dynamically adjusts the search region based on the target’s motion and environmental context, ensuring precise tracking even under challenging conditions such as occlusions, fast motion, and scale variations. By integrating an adaptive search mechanism, our SAMViTrack significantly reduces computational overhead without compromising accuracy, making it suitable for real-time deployment on UAVs with limited onboard resources. Extensive experiments on benchmark datasets demonstrate that our method outperforms both traditional and modern trackers, achieving superior accuracy and robustness with improved efficiency. The proposed tracker sets a new baseline, especially by combining Mamba and ViT, for UAV tracking by offering a balance between speed, accuracy, and adaptability in dynamic environments.

## 1. Introduction

The code for SAMViTrack is publicly available at https://github.com/open-at-25/SAMViTrack (accessed on 4 December 2025).

Unmanned Aerial Vehicles (UAVs) have become essential tools in a variety of applications, including surveillance [1], search and rescue [2], environmental monitoring [3], and package delivery [4]. A core challenge in these applications is achieving fast and reliable object tracking in real-time, as UAVs often operate in dynamic and unpredictable environments. Robust UAV tracking systems must handle rapid target movement, scale variations, occlusions, and environmental changes while maintaining high computational efficiency to meet real-time requirements [5,6,7,8].

Traditional tracking algorithms commonly use fixed search regions to locate a target in successive video frames. In these methods, a predefined area around the previously detected target is used as the search region to predict the target’s position in the next frame. While this approach is computationally efficient and straightforward to implement, it suffers from significant limitations in dynamic environments. One of the key issues with fixed search regions is their lack of adaptability to changes in the target’s movement or appearance. When the target moves unpredictably—such as sudden accelerations, sharp turns, or changes in direction—a static search region may fail to encompass the target’s new location [9,10]. This often results in tracking drift, where the tracker gradually loses sight of the target and begins to follow background objects instead [11,12,13]. Additionally, fixed search regions are vulnerable to environmental changes, such as occlusions, lighting variations, or cluttered backgrounds [14,15,16]. In real-world UAV tracking scenarios, targets can be partially or fully obscured by obstacles, making it difficult for a fixed search region to capture the target’s exact position. Furthermore, targets may change in size or shape due to variations in camera angles or distance, which a static search region cannot accommodate effectively. Recent advancements in deep learning-based trackers have improved tracking accuracy by leveraging powerful feature extraction models such as convolutional neural networks (CNNs) and transformers [5,17,18]. Among these, the Vision Transformer (ViT) [19] has shown significant promise due to its ability to capture both local and global context. However, the computational overhead of ViT due to the quadratic complexity of its attention mechanism makes it challenging for real-time UAV applications with limited onboard resources.

To address these challenges, we propose the Search-Region Adaptive Mamba-ViT Tracker (SAMViTrack), a novel framework designed to achieve fast and robust UAV tracking in real-world scenarios. The proposed tracker combines the efficiency of Mamba [20], a lightweight attention mechanism, with the powerful feature extraction capabilities of ViT to deliver both accuracy and speed. Combining Mamba and ViT leverages their complementary strengths to create an efficient and powerful tracking system. Mamba provides a lightweight attention mechanism that reduces computational costs, ensuring fast processing for real-time applications without sacrificing accuracy [21,22,23,24]. On the other hand, ViT excels at extracting rich, high-level features by capturing global dependencies across input data, which is essential for accurate tracking in complex scenarios [25,26]. The synergy between Mamba’s efficiency and ViT’s robust feature extraction strikes a balance between speed and accuracy, enhancing the tracker’s performance in dynamic environments. Together, they enable a system that is both fast and accurate, making it ideal for real-time tracking tasks. Another key innovation in our method is the adaptive search region mechanism, which dynamically adjusts the search area based on the target’s motion and environmental context. This adaptive mechanism allows the tracker to focus on the most relevant areas, reducing unnecessary computations and improving tracking performance in challenging conditions.

**Figure 1:**
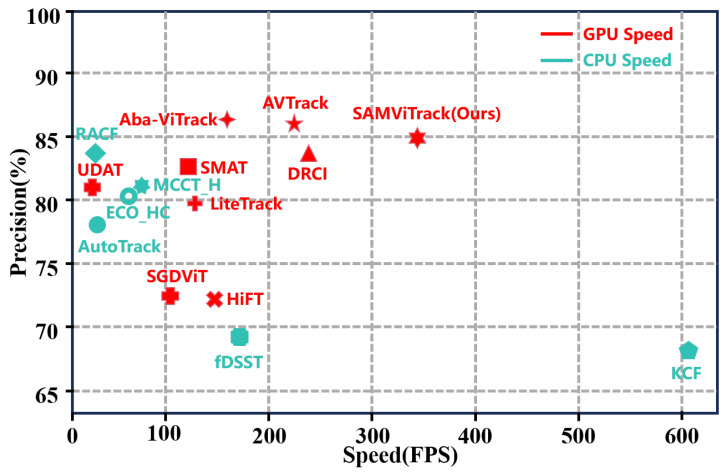
Compared with state-of-the-art UAV tracking algorithms on VisDrone2018, our SAMViTrack performs with 0.84 precision and still runs efficiently at approximately 340 FPS.

The proposed SAMViTrack framework addresses two critical needs in UAV tracking: (1) maintaining high tracking accuracy under diverse environmental conditions and (2) reducing computational complexity for real-time deployment. By integrating Mamba’s fast attention mechanism, SAMViTrack optimizes the traditional multi-head self-attention used in ViT, making it more efficient for resource-constrained UAV systems. Additionally, the adaptive search region mechanism enhances the tracker’s robustness by preventing target drift and improving recovery from occlusions and fast target movement. We evaluate our method on several benchmark datasets across various UAV tracking scenarios. The results demonstrate that SAMViTrack achieves superior performance compared to both traditional trackers and state-of-the-art deep learning models, with significant improvements in tracking accuracy and computational efficiency. As shown in Figure 1, our tracker achieves a superior balance between accuracy and efficiency, outperforming the leading tracker Aba-ViTrack by over 150 frames per second (FPS) while maintaining comparable precision. This makes it well-suited for deployment on UAV platforms with constrained computational resources. The main contributions of this paper are summarized as follows:

- A lightweight hybrid backbone integrating Mamba and ViT. We design a compact Mamba–ViT hybrid backbone that leverages Mamba’s efficient sequence modeling and ViT’s strong spatial representation. Unlike pure-Mamba or pure-VT variants, our hybrid design achieves a balanced trade-off between accuracy and computational overhead, making it suitable for UAV platforms with limited resources.
- A plug-and-play adaptive search-region mechanism. We introduce a search-region adaptive (SA) module that adjusts the size of the search area based solely on the relative velocity of the target and is activated only during inference. Unlike existing adaptive search mechanisms, such as Aba-ViTrack, which relies on auxiliary appearance cues and online regression, our method does not require additional network branches, online optimization, or extra model parameters. This makes the SA module architecture-agnostic, easily attachable to various trackers, and free of training-time cost.
- Extensive validation demonstrating strong generalization. Although designed for UAV tracking, the proposed SA module provides consistent improvements when integrated into different state-of-the-art trackers (e.g., OSTrack, AQATrack, HIPTrack) and evaluated on both UAV and general tracking benchmarks. These results confirm that the mechanism is not restricted to UAV viewpoints but serves as a broadly applicable dynamic search strategy.

## 2. Related Works

Visual Tracking. Existing visual tracking approaches can be broadly categorized into DCF-based trackers and deep learning (DL)-based trackers. Classical DCF trackers, such as KCF, ECO, and CSR-DCF, have been widely used in UAV scenarios due to their computational efficiency. UAV-oriented variants, including AutoTrack [27], HIFT [28], and ARCF [29], further improve robustness by incorporating adaptive filtering or handcrafted feature representations. However, these methods are still limited by their relatively weak feature expressiveness, making them sensitive to fast motion, occlusion, and significant appearance changes.

DL-based trackers, especially lightweight CNN-based approaches such as HiFT [30] and TCTrack [5], offer stronger robustness but generally exhibit higher computational cost. Recent efforts have explored model compression and dynamic inference strategies, such as rank-based pruning [6] and Fisher pruning [31], to reduce redundancy. More recently, ViT-based trackers have gained prominence due to their ability to capture long-range dependencies [32,33,34,35]. Among them, Aba-ViTrack [7] employs adaptive background-aware token selection to improve efficiency, while AVTrack [36] dynamically activates transformer blocks based on scene content. Despite these advances, the reliance on full transformer architectures and attention-heavy computation still presents challenges for real-time UAV deployment.

In contrast to existing approaches, our work integrates Mamba and ViT to balance efficient sequential modeling with strong global feature extraction, and introduces an inference-only adaptive search-region mechanism that requires no additional training or model branches. This distinguishes our method from prior adaptive transformer-based trackers and enables efficient deployment on resource-constrained UAV platforms.

In this paper, we propose a novel framework that integrates the efficiency of Mamba attention with the strong feature extraction capabilities of ViT, aiming to achieve an improved balance between efficiency and accuracy for UAV tracking.

Adaptive Mechanisms for Efficient Tracking. Adaptive mechanisms have emerged as a promising approach to achieving efficient tracking, particularly in real-time applications such as UAV tracking [7,36,37,38,39,40,41]. These methods focus on dynamically adjusting computational resources based on the complexity of the input, ensuring that the system allocates its resources optimally without sacrificing tracking performance. The goal is to strike a balance between computational efficiency and tracking accuracy, which is crucial for applications where both speed and precision are required. Aba-ViTrack [7] introduced an adaptive token-based approach to improve efficiency in real-time UAV tracking. AVTrack [36] and ABTrack [38] employed a more structured conditional computation strategy, where transformer blocks are adaptively activated or bypassed. BDTrack [37] dynamically exits transformer blocks during tracking, while DyTrack [42] presents a dynamic transformer framework that adjusts reasoning paths based on input complexity to optimize computational resources.

Although these adaptive strategies improve both efficiency and accuracy, they rely on learning adaptive feature extractors, which have some drawbacks. These extractors often need extra training or fine-tuning on diverse datasets, making model development more complex and time-consuming. The additional training and computational costs can reduce the expected efficiency gains. Moreover, these extractors require extra processing layers or architectures, increasing computational demands during inference. In this paper, we propose a novel adaptive strategy that improves performance by dynamically adjusting the search region based on the target’s motion and the surrounding environmental context. This plug-and-play module requires no additional training and can be seamlessly integrated into existing methods.

Vision Mamba Models. Traditional transformers require significant memory, especially for long sequences or large images, due to the quadratic growth of attention [43,44,45]. Mamba [20] addresses this by using an input-dependent selection mechanism and optimized parallel processing for efficient long-range dependency modeling. Originally designed for NLP, Mamba has quickly gained traction in vision tasks. Models like Vim and VMamba have set new benchmarks in image classification with advanced scanning mechanisms. It has also been applied to high-resolution tasks, such as medical image segmentation in VM-UNet [46] and Swin-UMamba [47]. Notably, MambaTrack has demonstrated its effectiveness in multi-object tracking, showcasing its ability to model complex motion and temporal relationships.

To our knowledge, however, combining Mamba and ViT for an efficient and powerful UAV tracking system has not been explored before. In this paper, we investigate how to leverage their complementary strengths to achieve a better balance between speed and accuracy for real-time UAV tracking. Hopefully, Mamba provides a lightweight attention mechanism that reduces computational costs, enabling fast tracking without sacrificing accuracy. Meanwhile, ViT captures global dependencies in the input data, extracting rich features essential for accurate tracking in complex scenarios.

Trade-offs in Complex Motion Modeling and Efficiency. Modeling the motion of intelligent agents in complex, multi-agent environments often requires capturing inter-agent interactions, intentions, and latent behavioral states, which are capabilities beyond simple kinematic cues. Game-theoretic and social interaction-based frameworks provide concrete methodological solutions for these challenges. For example, Social Interaction-Aware Dynamical Models [48] integrate social cues into coupled multi-agent dynamics, while asymmetric driving aggressiveness modeling [49] and uncertainty-aware estimation of social preferences [50] capture heterogeneous agent behaviors and quantify decision-making uncertainty. These approaches achieve strong intent- and interaction-aware anticipation but rely on deep recurrent networks or iterative optimization to infer high-dimensional states, resulting in substantial computational overhead.

In contrast, our SAMViTrack adopts a lightweight, reaction-based approach within the Single-Object Tracking paradigm. By using only the single-frame relative velocity $v_{\mathrm{rel}}\left(t-1\right)$ as the dynamic cue, it avoids multi-agent latent-state inference and high-order optimization. This trade-off places our approach at a fundamentally different point on the complexity–performance curve: we sacrifice long-horizon, intent-aware prediction for constant-time computation O(1), enabling real-time efficiency and plug-and-play usability suitable for resource-constrained UAV platforms.

## 3. Method

**Figure 2:**
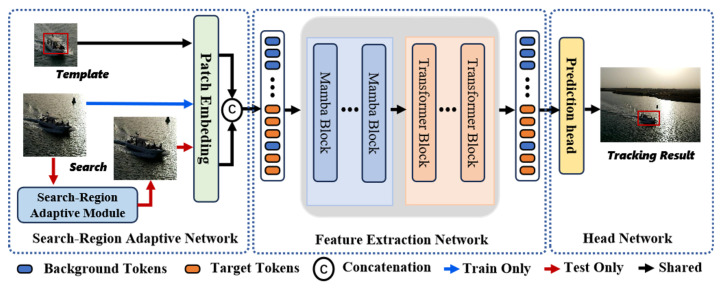
Overview of the proposed SAMViTrack framework. It consists of a Search-Region Adaptive Network, a Feature Extraction Network, and a Head Network. Note that the Search-Region Adaptive module is exclusively active during inference.

In this section, we introduce the proposed end-to-end tracking framework, SAMViTrack. This framework is designed to achieve efficient and accurate object tracking by combining the strengths of the Mamba model and ViT for feature representation, along with a mechanism that dynamically adjusts the search region size. The framework consists of three main components: the Search-Region Adaptive Network, the Feature Extraction Network, and the Head Network. An overview of the model is illustrated in Figure 2.

### 3.1. Overview

As illustrated in Figure 2, the proposed SAMViTrack framework adopts a single-stream architecture, which consists of a Search-Region Adaptive network, a backbone network, dubbed Mamba-ViT, based on Vision Mamba and Vision Transformer (ViT), and a tracking head. The framework takes a pair of images as input: the template image $Z\in R^{3\times H_{z}\times W_{z}}$ and the search image $X\in R^{3\times H_{x}\times W_{x}}$. These images are divided and flattened into sequences of patches with resolution P×P, resulting in $P_{z}=H_{z}\times W_{z}/P^{2}$ patches for Z and $P_{x}=H_{x}\times W_{x}/P^{2}$ patches for X. The features extracted from the Mamba-ViT backbone are then fed into the tracking head to generate the final tracking results. The Search-Region Adaptive Module is designed to dynamically adjust the size of the search region based on the target’s current relative speed. After the input template and search images undergo Patch Embedding, the resulting tokens are fed into the Feature Extraction Network, which consists of multiple Mamba and ViT layers. The integration of Mamba and ViT layers will be thoroughly examined in the Section 4.

### 3.2. Hybrid Mamba-ViT for Feature Representation

The proposed hybrid architecture leverages Mamba’s adaptive attention for efficiency and ViT’s global feature modeling to enhance representation power. This balance between efficiency and richness is essential for real-time UAV tracking. Our experiments show that placing ViT blocks after Mamba blocks yields better performance. More intricate combinations, such as alternating the stacking order, are beyond the scope of this work and will be explored in future studies. The details of the Mamba and ViT layers are provided below.

Vision Mamba for Tracking: In our proposed framework, the Vision Mamba module plays a crucial role in feature extraction for tracking tasks. Given the template image *Z* and the search image *X*, we first transform them into a sequence of one-dimensional tokens through a patch embedding process facilitated by a trainable linear projection layer. This results in K tokens, mathematically represented as:

(1) $$t_{1:K}^{0}=E\left(Z,X\right)\in R^{K\times E},$$

where *E* denotes the embedding dimension of each token. These input tokens $t_{1:K}^{0}$ are then fed into the encoding layer, where they undergo processing through $L_{m}$ stacked layers of bidirectional Vision Mamba (Vim) encoders [51]. Let $m^{l}$ represent the Mamba block at layer $l⩽L_{m}$, which processes all tokens from layer (l−1) via $t_{1:K}^{l}=m^{l}\left(t_{1:K}^{l-1}\right)+t_{1:K}^{l-1}$. Specifically, the input $t_{1:K}^{l-1}$ is initially normalized and subsequently processed through two distinct linear projection layers to yield intermediate features V and Q:

(2) $$\begin{array}{c} V={\mathrm{Linear}}^{v}\left(\mathrm{Norm}\left(t_{1:K}^{l-1}\right)\right),\\ Q={\mathrm{Linear}}^{q}\left(\mathrm{Norm}\left(t_{1:K}^{l-1}\right)\right). \end{array}$$

Although both are derived from the same input, they are generated via distinct linear projections (${\mathrm{Linear}}^{v}$ and ${\mathrm{Linear}}^{q}$), ensuring different parameterizations. In the Mamba block, Q acts as the input to the selection mechanism (determining content-dependent state transitions), while V provides the content that is processed by the SSM.

Next, V is processed in both forward and backward directions. The State Space Model (SSM) [20] captures the dynamic variations within a sequence by mapping the input sequence onto a latent state space. In each direction, a 1D convolution followed by a Sigmoid Linear Unit (SiLU) activation function [52] is applied to produce $V^{\prime }$:

(3) $$\begin{array}{c} V_{o}=\mathrm{SSM}\left(\mathrm{SiLU}\left(\mathrm{Conv}1d\left(V\right)\right)\right),\\ V_{o}^{\prime }=V_{o}⊙\mathrm{SiLU}\left(V\right),\\ Y\;\;=\mathrm{Linear}\left(V_{\mathrm{forward}}^{\prime }\right)+\mathrm{Linear}\left(V_{\mathrm{backward}}^{\prime }\right), \end{array}$$

where the subscript o indicates the two scan orientations. Bidirectional scanning facilitates interactions among all elements within the sequence, establishing a global and unconstrained receptive field. The output of the last layer $m^{L_{m}}$ is fed into the ViT encoder for further processing. Here, ⊙ denotes element-wise multiplication between matrices of the same dimension.

Vision Transformer for Tracking: The Vision Transformer (ViT) layer also serves as a key component for feature extraction and relationship modeling in our framework. The output tokens $t_{1:K}^{L_{m}}$ from the Mamba encoder are subsequently passed into the ViT encoder, which is composed of $L_{v}$ ViT layers. Specifically, each ViT layer consists of two main modules: Multi-Head Self-Attention and FFN. The Multi-Head Self-Attention mechanism is implemented as follows:

(4) $$\mathrm{Attention}\left(Q,K,V\right)=\mathrm{softmax}\left(\frac{QK^{⊤}}{\sqrt{d_{k}}}\right)V.$$

Here, *Q*, *K*, and *V* represent the Query, Key, and Value matrices obtained through linear transformations, respectively, and $d_{k}$ denotes the feature dimension of each attention head. The Multi-Head mechanism enhances the model’s representational capacity by partitioning the feature space into multiple subspaces:

(5) $$\mathrm{MultiHead}\left(Q,K,V\right)=\mathrm{Concat}\left({\mathrm{head}}_{1},\ldots ,{\mathrm{head}}_{M}\right)W^{O},$$

where *M* represents the number of attention heads, and $W^{O}$ is a learned projection matrix that combines the outputs of all attention heads into a unified representation. This mechanism allows the model to capture diverse relationships and dependencies within the input data, significantly improving its ability to handle complex patterns. The Feed-Forward Network (FFN) further processes the features, defined as:

(6) $$\mathrm{FFN}\left(x\right)=\max\left(0,W_{1}x+b_{1}\right)W_{2}+b_{2},$$

where *x* represents the input feature vector, $W_{1}$ and $W_{2}$ are weight matrices, $b_{1}$ and $b_{2}$ are bias terms, and max(·,·) is the ReLU activation function that introduces non-linearity. The FFN first transforms the input features into a higher-dimensional space and then projects them back, enabling the model to learn and capture complex patterns effectively. The output of each sublayer (self-attention and FFN) is normalized using Layer Normalization and combined with the input via residual connections: LayerNorm(x+Sublayer(x)). By stacking multiple ViT layers, the model progressively optimizes the extraction of target features and suppression of background noise, thereby achieving robust object tracking in complex scenarios.

### 3.3. Search-Region Adaptive Module

**Figure 3:**
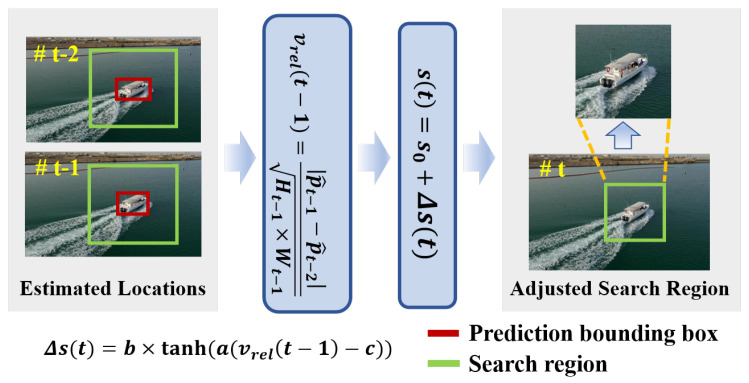
Illustration of the Search-Region Adaptive Module. The module takes two consecutive frames as input. Using relative velocity, the module computes the adaptive scaling factor (*s*) which is used to generate the adaptive search region.

The proposed Search-Region Adaptive Module dynamically adjusts the size of the search region based on the target’s motion to improve tracking accuracy and optimize computational efficiency. The workflow of the module is illustrated in Figure 3. Let the estimated location of the target at *t* frame be denoted by ${\hat{p}}_{t}$. Then the relative speed of the target at t−1 can be estimated by

(7) $$v_{\mathrm{rel}}\left(t-1\right)=\frac{\left|{\hat{p}}_{t-1}-{\hat{p}}_{t-2}\right|}{\sqrt{H_{t-1}\times W_{t-1}}},$$

where $\left(H_{t-1},W_{t-1}\right)$ denotes the predicted target size at t−1. Larger objects might move slower relative to their size, while smaller objects might have a higher relative speed despite moving at the same pixel speed. This calculation provides a measure of the target’s speed relative to its size, offering insights into how fast the object is moving in relation to the area it occupies in the frame. Intuitively, the search region should be smaller when the target moves slower and larger when the target moves faster. However, traditional trackers typically use a fixed scale factor, denoted by $s_{0}$, to determine the search region size without tacking this into account. In this paper, we extend this approach by introducing an adaptive scale factor, s(t), which adjusts based on the relative speed of the target, and is formulated by $s\left(t\right)=s_{0}+\Delta s\left(t\right)$, where

(8) $$\Delta s\left(t\right)=b\times \tanh\left(a(v_{\mathrm{rel}}\left(t-1\right)-c)\right),$$

where tanh(·) denotes the hyperbolic tangent function, and *a*, *b*, and *c* are predefined constants that regulate the sensitivity and magnitude of the scale adjustment. Specifically, the parameter *a* controls how strongly the scale variation responds to changes in relative velocity, with larger values producing more pronounced adjustments. The parameter *b* defines the maximum allowable change by constraining Δs to the bounded interval [−b,b], thereby preventing excessively large or small search regions. The parameter *c* serves as an offset that shifts the activation threshold of the adjustment, allowing the mechanism to better adapt to the motion characteristics of different targets. While this formulation models motion dynamics using a simplified relative-speed descriptor, such a design allows the adaptive mechanism to remain lightweight, inference-only, and fully plug-and-play, avoiding the additional computational overhead that more complex motion predictors typically introduce.

We emphasize that the efficiency gains of our method primarily arise from reducing the computational overhead of image resizing, rather than accelerating the model’s inference process. Since resizing is performed on every frame, its cumulative cost becomes non-negligible. The computational complexity of image resizing depends on both the interpolation method and the dimensions of the output image, and is typically linear in the number of output pixels. For instance, the bilinear interpolation used in this work has a complexity of $O(H^{\prime }\times W^{\prime })$, where $H^{\prime }$ and $W^{\prime }$ are the height and width of the resized image. However, because $H^{\prime }$ and $W^{\prime }$ are fixed input dimensions, the runtime efficiency is largely influenced by the overhead of data access, which in turn depends on the size of the search image prior to resizing. In the following, we provide a rationale for how our proposed method improves efficiency by adaptively adjusting this size. Denote the size of the search image prior to resizing as a two-dimensional discrete random variable H×W. Assuming the data access overhead is proportional to this size, the associated expected computational complexity of resizing the search image in our tracker can be expressed as:

(9) $$\begin{array}{c} E[O(H\times W)]=O(E(H\times W))\\ =O(E(H)\times E(W)+Cov(H,W)), \end{array}$$

where E is the expected value operator, Cov means covariance. It is evident that reducing E(H), E(W), or Cov(H,W), leads to a decrease in the expected computational complexity of the resizing operation. Existing methods typically use a fixed scale factor, commonly set to 4, which represents the ratio between the search area and the target area, to determine the size of the search image. In contrast, we adapt this factor by generalizing it into a function of the target’s relative speed, $v_{rel}$. When $v_{rel}$ falls below a predefined threshold *c* (as defined in Equation (8)), the scale factor is reduced, thereby decreasing E(H), E(W), which leads to improved computational efficiency. Conversely, when $v_{rel}$ exceeds *c*, the scale factor increases, expanding the search region and enhancing robustness against occlusions and rapid target motion—thus improving tracking accuracy. Experimental results demonstrate that, by appropriately setting the parameters of the scale factor function, our method is able to achieve simultaneous improvements in both efficiency and accuracy.

### 3.4. Tracking Head and Loss Function

In alignment with OSTrack [53], we implement a center-based head composed of multiple Conv-BN-ReLU layers to directly estimate the target’s bounding box. The head outputs local offsets to correct for discretization errors caused by resolution reduction, normalized bounding box sizes, and an object classification score map. The position with the highest classification score is selected as the object’s location, resulting in the final bounding box for the object. During training, we adopt the weighted focal loss for classification, a combination of $L_{1}$ loss and Generalized Intersection over Union (GIoU) loss for bounding box regression. The total loss function is defined as follows:

(10) $$L_{\mathrm{total}}=L_{\mathrm{cls}}+\lambda_{\mathrm{iou}}L_{\mathrm{iou}}+\lambda_{L_{1}}L_{L_{1}}.$$

where the trade-off parameters are set as $\lambda_{\mathrm{iou}}=2$ and $\lambda_{L_{1}}$ = 5 in our experiments.

Although the parameters *a*, *b*, and *c* are set empirically, their roles are simple and intuitive, and they follow common practice in lightweight adaptive mechanisms. The parameters mainly serve to keep the scale updates stable and predictable, preventing the search region from changing too quickly or excessively. Since the search-region adjustment is applied only during inference, learning these parameters through training would introduce additional complexity without providing clear benefits. Using fixed values therefore matches the design goal of the SA module, which is intended to be lightweight, plug-and-play, and independent of model retraining.

### 3.5. Parameter Sensitivity Observation

To further address concerns about the manually set parameters in the adaptive scaling function, we conducted a preliminary sensitivity study on the three parameters (a,b,c) across six evaluation settings (AQATrack–GOT10k, AQATrack–OTB, HIPTrack–GOT10k, HIPTrack–OTB, OSTrack–GOT10k, and OSTrack–OTB). All performance scores were normalized via min–max normalization to allow consistent comparison.

Overall, the tracker demonstrates stable behavior across a broad parameter range, and the performance curves vary smoothly without abrupt degradation. Importantly, all six model–dataset combinations exhibit consistent trends.

Parameter *a*. It controls the responsiveness of the hyperbolic tangent in Equation (8). Extremely large values (e.g., a>40) cause over-reactive scale changes and reduce stability. Across all six evaluation settings, the optimal region consistently lies within a∈[10,25], where the adaptive behavior remains both smooth and effective.

Parameter *b*. This parameter determines the maximum deviation from the base scale. The sensitivity curves show that nearly all models reach their best performance within a narrow and stable range of b∈[0.5,0.8]. This robustness suggests that *b* can be treated as a fixed constant in practice, without requiring per-model tuning, which greatly reduces hyperparameter dependency.

Parameter *c*. It functions as the relative-motion threshold. All six evaluation settings reveal an optimal region of c∈[0.9,1.2]. When *c* becomes too large (e.g., c≥1.8), the scale adjustment mechanism becomes overly conservative, leading to noticeable performance degradation.

These observations indicate that the proposed formulation is sufficiently robust for inference-only deployment, as performance varies smoothly and remains stable throughout practical parameter ranges. Nevertheless, obtaining a more comprehensive and theoretically unified understanding would require a substantially larger experimental grid across diverse target categories, motion patterns, and UAV environments—an exploration that falls beyond the scope of this work. Future research may incorporate a lightweight learnable module to automatically optimize (a,b,c) during training, providing a more principled and data-driven formulation.

### 3.6. Summary and Motivation for Adaptive Search

The adaptive search mechanism is motivated by the observation that the computational cost of transformer-based trackers is strongly influenced by the spatial size of the search region. A larger search area increases the number of tokens entering the backbone, thereby raising FLOPs, memory usage, and inference latency, while an overly small search region increases the risk of target drift. Our design aims to balance these two factors by dynamically adjusting the search region based on the estimated relative motion. This mechanism requires no retraining and incurs negligible overhead, making it suitable for real-time UAV tracking. The following section evaluates how this adaptive strategy affects tracking accuracy, robustness, and efficiency across several benchmarks.

## 4. Experiments

Before presenting the experimental results, we briefly outline how the evaluations relate to the design considerations introduced in Section 3. Since the proposed approach incorporates a lightweight hybrid backbone together with an adaptive search-region mechanism applied only during inference, the experiments are designed to examine the effectiveness of the backbone design, the influence of the adaptive mechanism on both accuracy and efficiency, and its ability to generalize across different trackers and datasets.

In this section, we conduct extensive experiments on four UAV tracking benchmarks: DTB70 [54], UAV123 [55], VisDrone2018 [56], UAVDT [57], and WebUAV3M [58]. All evaluation experiments are conducted on a PC equipped with i9-10850K processor (3.6 GHz), 16GB RAM and an NVIDIA TitanX GPU. To validate our approach, we compare it with state-of-the-art trackers, which are categorized into lightweight and deep learning-based models based on their architecture and computational needs.

### 4.1. Implementation Details

Model. We adopt the proposed Mamba-ViT as the backbone, which consists of a three-layer Mamba encoder and a three-layer ViT encoder for consideration of efficiency. The search-region adaptive module is applied only during inference, where it dynamically adjusts the search area based on the target’s relative velocity. During training, however, no dynamic resizing is used; a fixed search region is employed to maintain stable optimization and ensure compatibility with the OSTrack loss design. The head of our tracker consists of a stack of four Conv-BN-ReLU layers. The sizes of the template and search region are set to 128 × 128 and 256 × 256.

Training. We train the model using splits from the GOT-10k [59], LaSOT [60], COCO [61], and TrackingNet [62] datasets. The batch size is set to 32. For optimization, we use the AdamW optimizer [63] with a weight decay of $10^{-4}$ and an initial learning rate of $4\times 10^{-5}$ for the backbone. The model is trained for 300 epochs, processing 60,000 image pairs per epoch, and the learning rate is reduced by a factor of 10 after 240 epochs. The hidden dimension of the model is set to 192, and the number of attention heads is set to 3.

Inference. During inference, we apply a Hanning window penalty to incorporate positional priors, as is common in tracking practices [64]. Specifically, the classification map is multiplied by a Hanning window of the same size, and the bounding box with the highest score after multiplication is selected as the final tracking result.

### 4.2. Comparison with Lightweight Trackers

The evaluation results of our trackers and the competing lightweight trackers are presented in Table 1. As shown, our SAMViTrack demonstrate superior performance among all these trackers in terms of average (Avg.) precision (Prec.), success rate (Succ.) and speeds. On average, RACF achieves the highest precision (72.3%) and success rate (49.2%) among DCF-based trackers. DRCI attains the highest precision at 77.5%, while UDAT leads with the highest success rate of 58.1% among CNN-based trackers. Our SAMViTrack achieved the highest speed, surpassing the second-fastest DRCI by 2.8% in Prec. and 3.2% in Succ. AVTrack-SA and Aba-ViTrack-SA, integrated with the Search-Region Adaptive module (SA), enhance Prec. by 1.2% and 1.0%, respectively, while improving efficiency. Remarkably, Aba-ViTrack-SA achieves the highest Prec. (82.7%) and Succ. (62.8%), while AVTrack-SA ranks second in Prec. and third in Succ. This improvement in both tracking accuracy and efficiency can be attributed to the proposed Search-Region Adaptive module, which effectively narrows the search area when the target moves slowly, minimizing background interference and reducing computational overhead. It is worth noting that SAMViTrack achieves a GPU speed 1.7 times faster and a CPU speed 1.3 times faster than Aba-ViTrack-SA, showcasing its outstanding performance. This demonstrates an excellent balance between tracking accuracy and efficiency, highlighting the advantages of our method and confirming its effectiveness in delivering SOTA performance in UAV tracking.

### 4.3. Comparison with Deep Trackers

The proposed SAMViTrack is also compared with 14 deep trackers in Table 2, showing the Precision (Prec.), Success (Succ.), and GPU speed on the UAVDT dataset. SAMViTrack stands out by achieving the highest Prec. and the fastest GPU speed, demonstrating its competitiveness in both accuracy and speed. Notably, our method outperforms LiteTrack (second in Prec.) by 0.4% and SMAT (third in Prec.) by 1.2%, while being more than twice as fast in GPU speed. Although HIPTrack achieves the highest Succ., SAMViTrack is nearly ten times faster. Furthermore, while MixFormerV2 ranks second in speed, our method surpasses it by 24.2% in Prec. and 15.9% in Succ., further highlighting its superior balance of accuracy and efficiency.

### 4.4. Attribute-Based Evaluation

**Figure 4:**
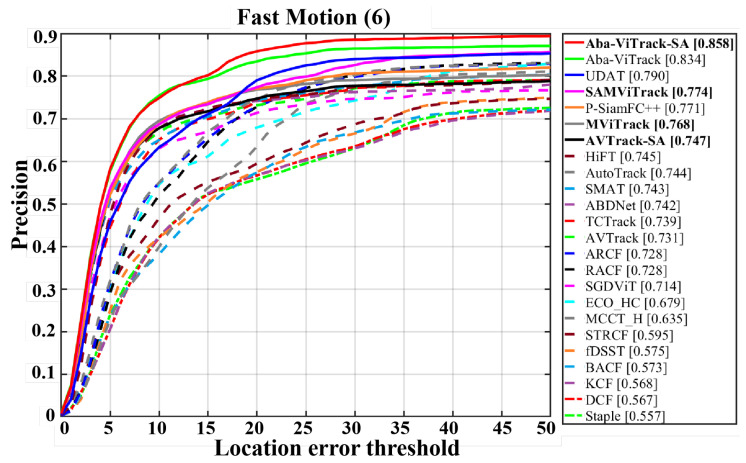
Attribute -based comparison on the fast motion subset of VisDrone2018. Note that MViTrack refers to SAMViTrack without utilizing the proposed adaptive search region mechanism.

To evaluate the effectiveness of the adaptive search region mechanism in addressing target fast motion, we conducted a comparative analysis of Aba-ViTrack-SA, AVTrack-SA, and SAMViTrack against 20 state-of-the-art (SOTA) trackers using the fast motion subset of the UAV123 dataset. Note that we also assessed SAMViTrack without applying the proposed method for the adaptive search region mechanism, denoted as MViTrack, for reference. The precision plot is illustrated in Figure 4. The results reveal that Aba-ViTrack-SA achieves the highest precision of 85.8%, ranking first among all evaluated trackers. Notably, the integration of the Search-Region Adaptive (SA) module improves precision by 2.4% compared to the original Aba-ViTrack. Similarly, AVTrack-SA and SAMViTrack demonstrate precision improvements of 1.6% and 0.6%, respectively, over their baseline versions. These findings highlight the crucial role of the adaptive search region mechanism in improving tracking performance by preventing target drift and enhancing recovery from occlusions and rapid target movement, especially in fast-motion scenarios.

### 4.5. Ablation Study

Impact of Mamba-ViT configurations and the Search-Region Adaptive module (SA). We conduct a thorough evaluation of both the backbone architecture configurations (i.e., the number of Mamba and ViT layers) and the effectiveness of our proposed Search-Region Adaptive (SA) mechanism on the UAVDT. Since there is no consensus on what constitutes the optimal balance between accuracy and efficiency, we define a measure, dubb $I_{b}$, to quantify the trade-off between these factors, which is based on the Efficacy Coefficient Method [88] and defined as follows:

(11) $$I_{b}\left(P,S\right)=\rho \mathrm{Ecoef}\left(P,P_{min}\right)+\left(1-\rho \right)\mathrm{Ecoef}\left(S,S_{min}\right),$$

where *P* and *S* denote the Prec. and the GPU speed, respectively, $P_{max}$ and $P_{min}$ are the maximum and minimum values of the data for normalization, $\mathrm{Ecoef}\left(P,P_{min}\right)=0.1+\frac{0.9\left(P-P_{min}\right)}{P_{max}-P_{min}}$ defines the Efficacy, ρ is the weight to trade-off precision and efficiency, which is set to 0.7 empirically. The experimental results are presented in Table 3. As can be seen, the Mamba-3/ViT-3 setting achieves an optimal balance between accuracy and efficiency, reaching the highest $I_{b}$ of 0.89, with a precision of 82.1%, a success rate of 58.1%, and a real-time inference speed of 346.2 FPS. Remarkably, the effectiveness of our proposed SA module is evidenced by consistent performance improvements across all configurations (denoted by ✓ in the SA column), with an average increase of 3.7% in precision and 1.9% in success rate while maintaining real-time processing capabilities. And the integration of SA with the Mamba-3/ViT-3 configuration also demonstrates superior performance, with gains of 2.8%, 1.3%, and 2.7% in Prec., Succ., and speed, respectively. These findings support our design for the SAMViTrack framework and highlight the effectiveness of our Search-Region Adaptive mechanism.

**Figure 5:**
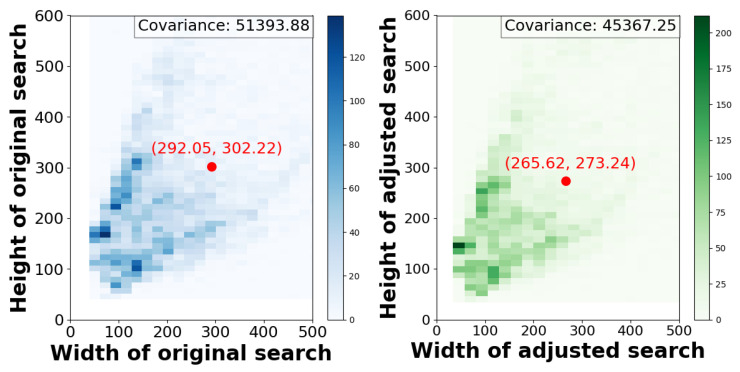
Comparison of two-dimensional histograms between the original and the adjusted search image.

In addition, to empirically validate that our SA effectively reduces the average size of the search image before resizing, we record the search dimensions at each frame of the DTB70 dataset, both with and without the application of SA. We then compare the changes using two-dimensional histograms of the original and adjusted search regions. As illustrated in Figure 5, the left panel shows the distribution of width and height for the original search regions, while the right panel displays the distribution after applying our SA. As observed, the adjusted search area exhibits a more concentrated distribution of width and height, indicating reduced covariance between the two dimensions. In fact, our method decreases both the average width and height. These findings qualitatively demonstrate that SA effectively reduces the average search image size before resizing, thereby lowering resizing overhead and validating its efficiency.

Evaluation of Mamba-ViT Configurations. In this experiment, we assessed the impact of various combinations of Mamba and ViT versions on the performance of deep learning models. The results in Table 4 indicate the configuration with the first three layers being Mamba and the last three layers being ViT demonstrated superior performance, achieving an accuracy of 82.1% and a success rate of 58.1%, along with the highest $I_{b}$ of 1.00. This configuration’s superior performance may stem from the Mamba layer’s effectiveness in capturing local features and the ViT layer’s proficiency in learning global features. The sequential processing of features from local to global may facilitate the ViT layer’s more effective integration of local features for global feature learning, thereby potentially enhancing the model’s overall representational capacity and generalization performance.

Furthermore, we added two pure baselines—6-layer Mamba and 6-layer ViT—for direct comparison. As shown in Table 4, both pure configurations underperform the hybrid models: pure Mamba lacks global modeling capacity, while pure ViT is less robust and less efficient. These results further confirm the complementary roles of Mamba and ViT and demonstrate that the hybrid design provides a more balanced and effective representation than using either architecture alone.

Application to SOTA Trackers. We evaluate the generalization ability of Search-Region Adaptative module (SA) by applying it to three state-of-the-art (SOTA) trackers: AQATrack [89], OSTrack-384 [53], and HIPTrack [79], on the GOT-10k [59], and OTB [90] datasets. As shown in Table 5, the SA module consistently improves tracking performance across all trackers. Notably, OSTrack experiences a significant boost with a 1.8% increase in SR0.75 on GOT-10k, while HIPTrack achieves a substantial gain of 1.3% in SR0.75. On the OTB dataset, all trackers show improvements of over 1.0% in Succ. and 0.8% in Prec. Additionally, the integration of SA consistently enhances GPU speeds. These results highlight the generalizability of the proposed SA module. While the module was initially developed for UAV tracking, its consistent improvements on GOT-10k and OTB show that it is not restricted to UAV-specific viewpoints and can serve as a broadly applicable dynamic search strategy across different tracking paradigms. Although the SA module is initially designed for UAV tracking tasks, our experimental results demonstrate its effectiveness in other tracking scenarios as well. However, due to limitations in time and resources, this study focuses solely on the application of UAV tracking for the time being.

Discussion on the 3 + 3 Configuration. As shown in Table 3 and Table 4, the configuration with the first three layers as Mamba and the last three layers as ViT achieves the most balanced performance. A plausible explanation is that the Mamba layers, which are efficient in modeling local dependencies, provide compact early representations, while the subsequent ViT layers benefit from these refined features to capture broader global context. This sequential progression from local to global information appears to form a stable and efficient feature hierarchy.

We note that this observation is based on the currently evaluated combinations. A full exploration of all possible layer permutations would require a much larger search space and extensive computational cost, which is beyond the scope of this work. Nevertheless, the existing results already indicate that moderately interleaving the two modules leads to stable performance without showing strong contradictory patterns. More systematic exploration of alternative hybridization strategies will be investigated in future work.

### 4.6. Computational Complexity

We provide a brief analysis of the computational cost of the proposed components. The hybrid Mamba–ViT backbone consists of three Mamba layers and three ViT layers. Mamba layers operate with linear complexity O(Ld), while ViT layers follow the standard quadratic self-attention complexity $O(L^{2}d)$, where *L* denotes the number of tokens and *d* is the embedding dimension. Replacing part of the transformer blocks with linear Mamba layers reduces the overall computation compared with a pure ViT backbone of comparable depth.

Impact on Feature Extraction Load. Although the adaptive search mechanism modifies the spatial crop size before feature extraction, it does not introduce additional branches or parameters. After cropping, the search region is resized to a fixed resolution and therefore fed into the backbone with an identical spatial size during inference. As a result, the FLOPs of the feature extraction network remain unchanged regardless of the expansion or contraction of the search region. The computational savings arise primarily from reducing unnecessary scaling operations when the target exhibits slow or smooth motion, rather than from altering the backbone complexity. The Search-Region Adaptive (SA) module introduces negligible overhead, as it updates the search size using only a scalar function defined in Equation (8), which has constant-time complexity O(1) and does not require additional feature extraction or attention computation. Overall, the tracker maintains a favorable computational profile, fully aligning with the empirical runtime improvements observed in Section 4.

### 4.7. Qualitative Results

**Figure 6:**
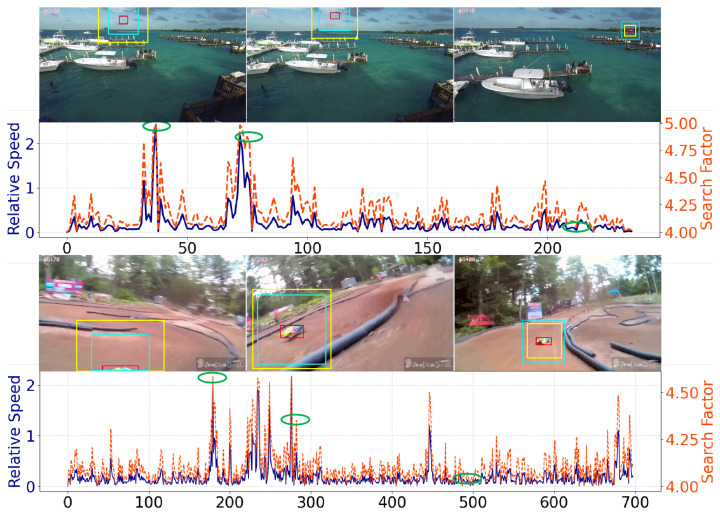
Illustration of the Search-Region Adaptive (SA) mechanism. The *x*-axis represents the frame index of a video sequence. The green-circled points correspond to the specific frames from which the visual examples (shown above) are taken. The red boxes indicate the true target location, the cyan boxes denote the fixed search region used in conventional methods, and the yellow boxes represent the adaptively adjusted search region.

Figure 6 shows two example sequences from DTB70 [54] (Gull2 and RcCar4) using adaptive search regions based on the target’s relative speed with the proposed SAMViTrack method. The red boxes highlight the target location, while the yellow and cyan boxes indicate the traditional fixed search region and our adaptively adjusted search region, respectively. The darkblue and tangerine curves represent the relative speed of the target and the corresponding adaptive search factor *s* produced by our method, where the search factor directly controls the degree to which the search region expands or contracts according to the target’s motion. The frames are marked with ellipses in the curves to indicate their time stamps. As shown, our method effectively expands the search area during fast target motion and contracts it during slower movements, successfully tracking both objects. This further supports the effectiveness of our Search-Region Adaptive (SA) mechanism in UAV tracking.

**Figure 7:**
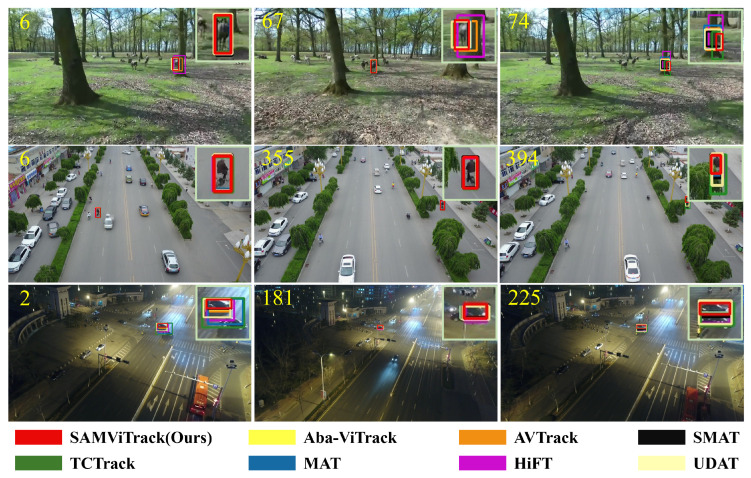
Qualitative comparison on three video sequences, respectively, from the datasets DTB70, VisDrone2018, and UAVDT (i.e., Horse2, uav0000353_01127_s, and So302). Each number indicates which frame of the video is displayed.

Figure 7 compares the tracking results of SAMViTrack with seven state-of-the-art trackers in three challenging scenarios. Our approach demonstrates remarkable robustness by maintaining accurate tracking across all test cases, while other methods encounter difficulties. Specifically, SAMViTrack excels in handling complex situations such as background clutter (e.g., Horse2 and So302), full occlusion (e.g., uav0000353_01127_s), and significant viewpoint changes. The consistent performance of SAMViTrack in these demanding conditions further highlights the effectiveness of our proposed UAV tracking framework.

## 5. Discussion

### 5.1. Limitations and Design Trade-Offs

Although SAMViTrack demonstrates strong performance across multiple UAV benchmarks, several limitations remain that merit attention.

Handling Abrupt Motion and Extreme Conditions. The proposed adaptive search mechanism is designed as a plug-and-play module that can be integrated into existing trackers without retraining. It adjusts the search region size based on the estimated relative motion from the previous frame, $v_{\mathrm{rel}}\left(t-1\right)$, enabling the region to contract during slow motion and expand when the target moves rapidly. However, because the update relies on motion cues from the preceding frame, this design introduces an inherent one-frame response delay. While such delay is negligible under smooth motion, it may affect responsiveness when the target undergoes abrupt acceleration or abrupt direction changes. In these cases, the contracted search region may not expand quickly enough, potentially causing temporary tracking failure if the target jumps outside the reduced region. Although the bounded tanh-based scaling stabilizes the update by constraining Δs within [−b,b], this also limits the maximum expansion rate and therefore the ability to handle extreme motion variations or momentary disappearances. These failure risks become more pronounced under severe motion blur or full occlusion, where short-term motion predictions become unreliable.

Multi-Target Scenarios. SAMViTrack follows the Single-Object Tracking (SOT) paradigm, where the tracker maintains a single adaptive search region for the target being tracked. As a result, the current formulation does not incorporate inter-target reasoning mechanisms, such as occlusion handling or cross-target interaction modeling. While a straightforward multi-target application (processing instances independently) is computationally feasible, the lack of these reasoning components limits the tracker’s ability to handle the complex, interacting behaviors and occlusions commonly observed in dense multi-UAV environments.

Complex Motion Modeling. One limitation of our current design is the coarse binary categorization of target motion (fast vs. slow). This simplification, intended to preserve the lightweight and plug-and-play characteristics of the adaptive search mechanism, sacrifices long-horizon, intent-aware prediction for computational efficiency.

Our approach is inherently reactive, using only the single-frame relative velocity $v_{\mathrm{rel}}\left(t-1\right)$ as the dynamic cue, resulting in a constant-time O(1) overhead for search adjustments. Unlike Trajectron++ [91] or Social-LSTM [92], which explicitly model multi-dimensional latent states and social interaction cues, our mechanism avoids recurrent inference or multimodal trajectory prediction. Similarly, unlike interactive decision frameworks [48,49,50], which estimate continuous aggressiveness parameters, asymmetric preferences, and uncertainty in social intent, our formulation reduces dynamics to a single reactive scalar. This deliberate trade-off limits predictive expressiveness but ensures real-time efficiency and plug-and-play usability on resource-constrained UAV platforms, without requiring additional training, deep network inference, or complex state-estimation solvers.

### 5.2. Future Directions

Integrating Anticipatory Dynamics. The limitations of the current reactive mechanism suggest a promising future direction: incorporating anticipatory dynamics inspired by specific socially interactive and game-theoretic models. For instance, integrating the asymmetric agent preference modeling approach from Socially Game-Theoretic Lane-Change [49] could enable intent-aware acceleration scaling, allowing the search region to expand preemptively in response to inferred adversarial maneuvers. Likewise, leveraging uncertainty quantification techniques from probabilistic forecasting frameworks such as Trajectron++ [91] or models for reducing social preference uncertainty [50] could allow the search region to adapt based on predicted outcome variance, making the mechanism risk-aware. Integrating these concrete strategies into the adaptive scaling function may enhance robustness under complex, interactive UAV scenarios while aiming to retain computational efficiency.

Multi-Target Extension. Extending SAMViTrack to handle multiple targets represents a promising direction for future work. Such an extension could explore strategies for maintaining independent search regions and adaptive scaling for each target, while leveraging the Mamba-ViT backbone for robust feature association and identity maintenance. Incorporating inter-target reasoning mechanisms, such as occlusion handling and cross-target interactions, may further enhance tracking robustness in complex multi-UAV environments.

Training with UAV-Specific Data. While SAMViTrack is trained on general large-scale tracking datasets (e.g., GOT-10k, LaSOT), these datasets exhibit viewpoint distributions that differ from typical UAV scenarios. UAV-specific datasets such as WebUAV-3M provide richer aerial-view variations and could potentially further improve viewpoint robustness for both the backbone network and the adaptive search module. Conducting such full-scale training or fine-tuning requires substantial computational effort, and is therefore left as an important direction for future work.

## 6. Conclusions

In this paper, we presented SAMViTrack, a novel framework that successfully combines Vision Mamba with Vision Transformers for real-time UAV tracking for the first time. By introducing an adaptive search region mechanism and integrating Mamba’s efficient attention computation and ViT’s strong feature extraction capabilities, we effectively addressed the dual challenges of maintaining high tracking accuracy while reducing computational overhead for real-time UAV applications. The dynamic search region adjustment enables robust tracking under challenging conditions such as occlusions, fast motion, and varying scales. Moreover, the modular nature of our approach allows for easy integration with existing tracking systems. Extensive experiments on multiple benchmark datasets show that our method delivers state-of-the-art performance while maintaining real-time processing capabilities on resource-limited UAV platforms. We believe SAMViTrack represents a significant step forward in real-time UAV tracking where computational efficiency is crucial and provides a solid foundation for future research in this domain.

## Figures and Tables

**Table 1:** Comparison of precision (Prec.), success rate (Succ.), speed (FPS), FLOPs and Param between SAMViTrack and lightweight trackers on DTB70, UAVDT, VisDrone2018, UAV123 and WebUAV-3M. **Red**, **blue**, and **green** signify the first, second, and third places. Please note that the percent symbol (%) is excluded for Prec. and Succ. values.

Tracker		Source	DTB70		UAVDT		VisDrone2018		UAV123		WebUAV-3M		Avg.		Avg. FPS		FLOPs	Param
			Prec.	Succ.	Prec.	Succ.	Prec.	Succ.	Prec.	Succ.	Prec.	Succ.	Prec.	Succ.	GPU	CPU	(GMac)	(M)
DCF-based	KCF [65]	TPAMI 15	46.8	28.0	57.1	29.0	68.5	41.3	52.3	33.1	39.8	21.6	52.9	30.6	-	**468.5**	-	-
	fDSST [66]	TPAMI 17	53.4	35.7	66.6	38.3	69.8	51.0	58.3	40.5	43.5	28.5	58.3	38.8	-	**132.4**	-	-
	ECO_HC [67]	CVPR 17	63.5	44.8	69.4	41.6	80.8	58.1	71.0	49.6	61.3	40.2	69.2	46.9	-	**66.9**	-	-
	MCCT_H [68]	CVPR 18	60.4	40.5	66.8	40.2	80.3	56.7	65.9	45.7	52.1	34.3	65.1	43.5	-	55.3	-	-
	AutoTrack [27]	CVPR 20	71.6	47.8	71.8	45.0	78.8	57.3	68.9	47.2	56.5	35.6	69.5	46.6	-	50.7	-	-
	RACF [28]	PR 20	72.6	50.5	77.3	49.4	83.4	60.0	70.2	47.7	58.0	38.4	72.3	49.2	-	33.2	-	-
CNN-based	HiFT [30]	ICCV 21	80.2	59.4	65.2	47.5	71.9	52.6	78.7	59.0	60.9	45.8	71.4	52.9	157.2	-	7.2	9.9
	UDAT [69]	CVPR 22	80.6	61.8	80.1	59.2	81.6	61.9	76.1	59.0	64.8	48.7	76.6	58.1	31.2	-	-	-
	TCTrack [5]	CVPR 22	81.2	62.2	72.5	53.0	79.9	59.4	80.0	60.5	61.9	45.7	75.1	56.2	132.7	-	8.8	9.7
	DRCI [70]	ICME 23	81.4	61.8	84.0	59.0	83.4	60.0	76.7	59.7	62.0	47.6	77.5	57.6	**268.5**	61.0	3.6	8.8
	SGDViT [71]	ICRA 23	78.5	60.4	65.7	48.0	72.1	52.1	75.4	57.5	61.3	45.7	70.6	52.7	107.3	-	11.3	23.3
	ABDNet [72]	RAL 23	76.8	59.6	75.5	55.3	75.0	57.2	79.3	60.7	63.9	48.7	74.1	56.3	119.2	-	-	-
Vit-based	Aba-ViTrack [7]	ICCV 23	**85.9**	**66.4**	**82.9**	**59.1**	**86.1**	**65.3**	**86.4**	66.4	67.4	53.7	**81.7**	**62.2**	172.1	46.3	2.4	8.0
	AVTrack [36]	ICML 24	84.3	**65.0**	82.1	58.7	86.0	**65.3**	84.8	**66.8**	69.0	54.0	81.2	61.9	252.8	53.8	0.97–1.9	3.5–7.9
	LiteTrack [73]	ICRA 24	82.5	63.9	81.6	**59.3**	79.7	61.4	84.2	65.9	69.4	54.1	79.5	60.9	132.6	-	14.1	54.92
	SMAT [74]	WACV 24	81.9	63.8	80.8	58.7	82.5	63.4	81.8	64.6	68.9	53.9	79.2	60.9	118.5	-	3.2	8.6
	Aba-ViTrack-SA		**86.1**	**65.2**	**83.1**	**59.4**	**87.7**	**65.7**	**86.0**	**67.9**	**70.7**	**55.5**	**82.7**	**62.8**	188.4	47.9	2.4	8.0
	AVTrack-SA		**85.5**	64.8	**82.8**	58.3	**86.6**	**65.4**	**85.8**	**67.2**	**71.2**	**55.3**	**82.4**	**62.2**	**268.0**	55.3	0.97–1.9	3.5–7.9
	SAMViTrack	Ours	83.3	63.8	82.1	58.1	84.1	63.6	81.9	64.1	**70.2**	**54.6**	80.3	60.8	**314.1**	61.6	1.1	4.0

**Table 2:** Precision (Prec.), success (Succ.), and GPU speed comparison between SAMViTrack and DL-based tracker on UAVDT. **Red**, **blue**, and **green** signify the first, second, and third places.

Tracker	Prec.	Succ.	FPS	Tracker	Prec.	Succ.	FPS	Tracker	Prec.	Succ.	FPS
SAMViTrack (Ours)	**82.1**	58.1	**346.2**	MixFormerV2 [33]	57.8	42.1	**248.0**	MAT [75]	72.9	54.6	71.2
DCPT [76]	76.8	56.8	41.2	ARTrack [77]	74.7	52.8	84.9	CSWinTT [78]	67.3	51.2	32.6
HIPTrack [79]	79.6	**60.9**	33.0	DropTrack [80]	78.8	57.0	**142.8**	SimTrack [81]	76.5	57.2	76.0
EVPTrack [82]	**80.1**	**60.3**	68.3	SeqTrack [83]	79.0	60.0	12.1	STARK [84]	72.0	53.6	53.3
ZoomTrack [85]	77.1	58.0	62.3	ROMTrack [86]	**81.9**	**61.6**	55.8	SiamGAT [87]	76.4	58.9	86.5

**Table 3:** Impact of Mamba-ViT configurations and Search-Region Adaptive (SA) on the tracking performance of SAMViTrack on the UAVDT benchmark. Here, ✓ indicates the addition of the SA module, and ↑ shows the improvement; for FPS, it represents the percentage increase relative to the original value.

Mamba Layers	ViT Layers	SA	Prec.	Succ.	FPS	*I_b_*
6	0		75.28	54.66	366.72	0.49
		✓	$80.24_{↑4.96}$	$56.95_{↑2.29}$	$377.7_{↑3.0\%}$	0.87
5	1		72.9	52.7	357.9	0.30
		✓	$79.7_{↑6.8}$	$57.1_{↑4.4}$	$367.9_{↑2.8\%}$	0.80
4	2		78.8	56.1	341.0	0.64
		✓	$80.5_{↑1.7}$	$58.9_{↑2.8}$	$351.4_{↑1.3\%}$	0.79
3	3		79.3	56.8	337.3	0.66
		✓	$82.1_{↑2.8}$	$58.1_{↑1.3}$	$346.2_{↑2.7\%}$	**0.89**
2	4		78.2	56.3	319.9	0.53
		✓	$79.4_{↑1.2}$	$56.6_{↑0.3}$	$326.2_{↑2.0\%}$	0.63
1	5		76.8	56.1	315.1	0.41
		✓	$80.3_{↑3.5}$	$57.0_{↑0.9}$	$324.8_{↑3.1\%}$	0.69
0	6		75.68	54.57	302.7	0.30
		✓	$80.63_{↑4.95}$	$55.83_{↑1.26}$	$313.9_{↑3.70\%}$	0.67

**Table 4:** Comparison of different Mamba and ViT combinations on the UAVDT benchmark. It presents Prec., Succ., FPS, and $I_{b}$ of the model under various combinations of Mamba and ViT, with and without SA enabled. Here, ✓ indicates the addition of the SA module.

Mamba	ViT	SA	Prec.	Succ.	FPS	*I_b_*
1, 3, 5	2, 4, 6		65.4	46.5	285.1	0.14
		✓	71.9	48.7	292.9	0.41
2, 4, 6	1, 3, 5		67.0	48.4	273.9	0.16
		✓	74.2	51.4	293.0	0.50
4, 5, 6	1, 2, 3		79.6	55.0	335.9	0.87
		✓	80.7	57.4	344.7	0.94
1, 2, 3	4, 5, 6		79.3	56.8	337.3	0.86
		✓	**82.1**	**58.1**	**346.2**	**1.00**

**Table 5:** Evaluation fany of the generalizability of our SA by applying them to three SOTA trackers. The evaluation is performed on GOT-10k and OTB. Here, ✓ indicates the addition of the SA module.

Tracker	SA	GOT-10k		OTB		Avg. FPS
		AO	SR0.75	Succ.	Prec.	
AQATrack [89]		76.0	74.9	68.8	89.8	22.6
	✓	**76.7**	**75.7**	**69.9**	**91.7**	**24.3**
OSTrack [34]		73.7	70.8	68.2	88.7	33.5
	✓	**74.7**	**72.6**	**69.4**	**90.5**	**35.2**
HIPTrack [79]		77.4	74.5	70.9	93.1	28.7
	✓	**78.4**	**75.8**	**71.7**	**93.9**	**30.0**

## Data Availability

Data is contained within the article.
